# Influence of Receptor Antagonists, Local Anesthetics, and Denervation on Microcirculation

**Published:** 2011-01-20

**Authors:** Heinz H. Homann, Tobias Hirsch, H.U. Steinau, Thomas Muehlberger, Wibke Moll, Marcus Lehnhardt, Ole Goertz

**Affiliations:** ^a^Department of Plastic, Reconstructive and Aesthetic Surgery, Hand Surgery, Helios-Klinikum Wuppertal, University of Witten/Herdecke, Germany; ^b^BG-University Hospital Bergmannsheil, Department of Plastic and Hand Surgery, Burn Center, Ruhr-University Bochum, Germany; ^c^Plastic Surgery/Migraine Surgery, DRK-Hospital Berlin, Germany; ^d^BG-Hospital Ludwigshafen, Department of Hand-, Plastic- and Reconstructive Surgery, Burn Center, University of Heidelberg, Germany

## Abstract

**Objective:** Impaired microcirculation is one of the most important factors in delayed wound healing. The aim of the study was to investigate the influence of chemical and surgical interruption of sympathetic nerve fibers and α- and β-receptors blockers on muscular microcirculation. **Methods:** The experiment was performed on a standardized cremaster muscle model of male Wistar rats (*n*=51). Microcirculation was recorded via transillumination microscopy on each of the 4 test groups and in a control group before and after their respective treatments with one of the following: topical application of bupivacaine, metoprolol, phentolamine, or surgical denervation. The arteriolar diameter and functional capillary density (FCD) as parameter for tissue perfusion were assessed. **Results:** The α-blocker phentolamine was the only agent that caused a significant dilation of the arteriolar diameter (76.6 ± 6.9 vs 100.0 ± 12.0 µm). However, like bupivacaine, metoprolol, and the surgical sympathectomy, it did not improve FCD as a parameter for tissue perfusion. The strongest vasoconstriction (35.9 ± 4.3 vs 28.6 ± 4.0) and impairment of the FCD (10.0 ± 0.7 vs 4.1 ± 0.9) was induced by the β-blocker metoprolol. **Conclusions:** This study shows that phentolamine could be an agent for dilating arteriolar diameter, but it did not improve FCD. Whereas the other agents, including sympathectomy, did not alter arteriolar diameter, the β-blocker worsened both investigated parameters. Our results raise the question whether β-blockers negatively influence microcirculation. Therefore, further studies are needed to investigate the potential adverse effects of β-blockers on wound healing.

Wound healing is a process involving complex cellular and extracellular mechanisms. It is well-known that wound healing depends mainly on tissue perfusion, and more specifically, on microcirculation. If the microcirculation is impaired, for example, by diabetes mellitus, radiation therapy, bacterial infection, or peripheral arterial occlusive disease, wound healing is impaired.^1^ It is therefore of the utmost importance that wound healing will not be further inhibited by any agent, and it would be desirable if topically applied agents could improve microcirculation.

In addition to metabolic influences, myogenic factors are responsible for the regulation of the vessel diameter in muscles and therefore for muscle perfusion.^2^ Precapillary sphincter systems control the capillary perfusion. The myogenic regulation is mediated by α- and β-receptors. The α_1_-receptors with their subtypes regulate primarily the first-order arterioles, whereas the α_2_-receptors regulate the third-order arterioles.^3^^-^6

Because many patients suffer from heart and blood pressure diseases, β-blockers are widely used. These same patients also often suffer from chronic dermal ulcers. β-blockers could be one factor in decreasing peripheral perfusion and, therefore, could impair wound healing.

There is also the question of whether the clinically used medical or surgical sympathectomy (Raynaud disease, arterial occlusive disease) alter arteriolar diameter and functional capillary density (FCD).^7^^-^10

The aim of our study was to investigate the effect of topically applied α- and β-receptor blockers, local anesthetics, and denervation on the muscular microcirculation in a rat model. An applied drug or intervention causing an increase of the arteriolar diameter and functional vessel density could be used in combination with antibacterial agents for treatment of critical wounds. Conversely, a drug causing decrease of these parameters should be avoided.

## METHODS

### Animals

The cremaster muscle of male Wistar rats were the subject of the study (*n*=51, 170-200 g; Harlan & Winkelmann, Borchen, Germany). The experiments were conducted in accordance with the National Research Council's guide for the care and use of laboratory animals.

### Anesthesia

Animals were anesthetized by intraperitoneally injected sodium pentobarbital (Narcoren, 50 mg/kg body weight; Merial GmbH, Hallbergmoos, Germany). If necessary, /one third of the initial dose was injected to maintain anesthesia. The animals were placed on a heated observation platform (Effenberger, Pfaffing, Germany), rectal temperature, and surface temperature of the prepared muscle were measured throughout the whole study period by using a 2-probe thermometer (Atkins Technical Inc, Mod 39658T, Gainesville, Florida).

After shaving the throat, the left carotid artery was prepared and a probe was inserted to monitor the blood pressure continuously throughout the study (Intramedic Polyethylene Tubing, PE-50, Becton Dickson, Parippany, NY; blood pressure meter: Monitor BP-1, World Precision Instruments, Berlin). To stabilize the blood pressure, saline solution was injected through the same catheter. A tracheotomy was performed to keep the airway open using tubing (Venofix, Luer Lock 21G, 0.8 × 2.0 mm, Braun, Melsungen, Germany), while animals breathed spontaneously.

### Cremaster Preparation

**Figure 1:**
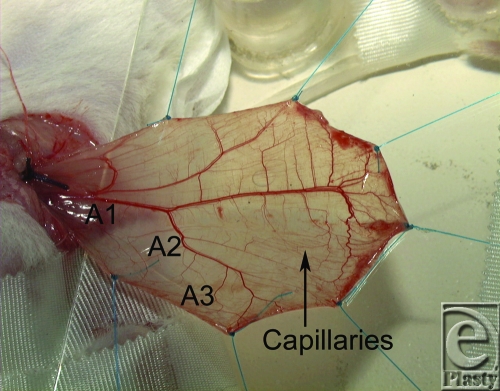
Cremaster muscle of the rat extended by 7 loops on the underlying glass slide. A_1_ marks the main cremaster feeding vessel, A_2_-arterioles branch off the main arteriole, and A_3_ branch off the A_2_-arterioles.

The model was first described by Baez et al.^11^ After isolating of the cremaster muscle, it was stretched using 7 loops on a specially designed observation platform (Fig 1), where the temperature was monitored.^12^

### Test solution and study groups

The α_1_-receptors were blocked by the topical application of phentolamine hydrochloride (*n*=8; Sigma Chemicals, Seelze, Germany), which was dissolved in aqua purificata (20 mg/mL), resulting in a concentration of 5 mg/cm^2^ at the cremaster muscle.^13^,^14^ The β-receptors were blocked by metoprolol tartrate (*n*=8; 3 mg, Beloc, Astra, Wedel, Germany).

The sympathetic fibers were suppressed by topical application of bupivacaine (*n*=8; 0.5%, 15 mg bupivacaine hydrochloride; Carbostesin, Astra, Wedel, Germany).

The denervation (*n*=15) was performed at the proximal cremaster muscle, where the periarteriolar tissue, including the nerves, were dissected.^15^

Saline solution (*n*=12; Natriumchlorid, B. Braun Melsungen AG, Melsungen, Germany) was used for control.

A volume of 3 mL of the solutions was applied to the surface of the muscle for 30 minutes and covered by a piece of self-adherent polyurethane dressing (OpSite? Dressing Film, Smith and Nephew Wound Management, Largo, Florida) to prevent the muscle for drying out and to keep the solution on the muscle. After 30 minutes, the solutions were rinsed out with saline solution.

### Exclusion criteria

If arterial blood pressure dropped below 80 mm Hg or exceeded 120 mm Hg even after intervention or if rectal body temperature (34.5°C-37.5°C) and temperature of the cremaster muscle surface (31.0°C-34.0°C) departed from the determined values, the animals were excluded from the study.

### Microscopy and recordings

For the transillumination microscopy (Axiotech vario, Carl Zeiss, Oberkochen, Germany) and recording, a charge-coupled video camera was used (Model Nr MC-3309, AVT-Horn, Aalen, Germany) and the recordings stored on super VHS videotapes (Panasonic AG-7350). Microscopic observations were performed before application of agents as well as 60 and 120 minutes after. Altogether 9 visual fields with a dimension of 0.55 to 0.40 mm were investigated.

We used a 10-fold water immersion objective (Achroplan 4x/20x, Zeiss), resulting in a total magnification of about 100-fold. Photographs were taken to ensure accurate relocation of the investigated areas throughout the study.

The diameter of the first (A_1_)-, second (A_2_)-, and third-order (A_3_) arteries in µm, as well as the perfused capillaries per area (0.22 mm^2^), was measured. After concluding the experiments, the animals were euthanized by an overdose of pentobarbital.

### Statistics

The commercially available computer program SPSS version 18 (SPSS GmbH, Munich, Germany) was used for statistical analysis of the data. The data collection of the recordings was blinded; the researcher did not know the interval of time between recordings or the agent being tested. Out of the single data, the mean value, the standard error, and the standard error of the mean of each animal were calculated. To compare the different values with each other, a variance analysis for repeated measurements was used. The mean value of the significant data was compared with the *t* test for paired samples. A *P* < .05 was considered statistically significant.

## RESULTS

The cremaster model in rats allows for good-quality recordings of arteriolar diameter and capillary function (Fig 1). In none of the animals, the core and muscle temperature or blood pressure exceeded or dropped below the determined values (Table 1).

**Figure 2:**
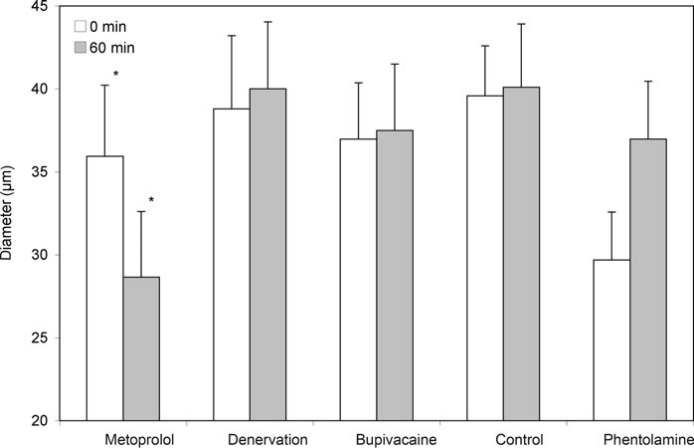
Mean diameter and SEM of third-order arterioles (A_3_). Recordings were performed before and 60 minutes after topical drug administration and denervation. Again, the phentolamine group showed the highest increase. The greatest decrease of arteriolar diameter was again found in the metoprolol group (*P < .05 vs baseline value; data is given in mean and SEM).

**Figure 3:**
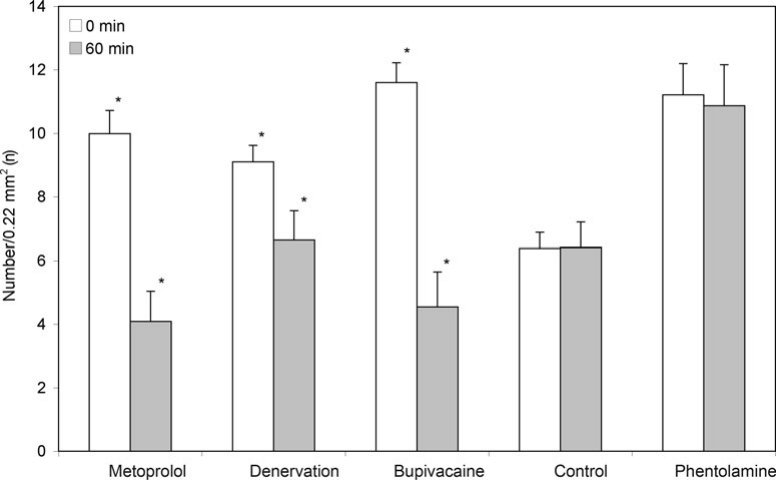
The functional vessel density of the 5 groups. A significant decrease in the density, as compared to baseline values, can be seen within 60 minutes after denervation and topical application of bupivacaine and metoprolol. Data is given as mean and standard error of mean (*P < .05 vs baseline value).

For the control group, no alterations of arteriolar diameter or FCD could be found at any of the measured points in time (Figs 2-5, Table 2).

The denervation of the sympathetic fibers caused a vasodilation, which occurred immediately after surgical sectioning and lasted for 30 minutes. After 60 minutes, we saw a decrease of the arteriolar diameter in A_1_- and A_2_-arterioles but no alterations in A_3_-arterioles (Table 2, Fig 2). The FCD decreased significantly (Table 2, Fig 3).The local anesthetic bupivacaine also caused a decrease of A_1_- and A_2_-arterioles and showed no influence on A_3_-arterioles.

The blockade of the β-receptors by metoprolol caused a decrease in arteriolar diameter in all arteriolar orders and caused the last-order arterioles to differ significantly from the baseline value. In addition, a significant decrease of the FCD could be found.

After the blockade of α_1_-receptors by phentolamine, an increase in arteriolar diameter was noticed in A_1_, A_2_, and A_3_-arterioles, but the values did not differ significantly from the baseline value. The FCD was not influenced by phentolamine.

The blockade of β-receptors by metoprolol led to a decrease in arteriolar diameter, with the values of the third-order arterioles differing significantly (*P* < .05) from those of the control animals (Table 2, Fig 2). In addition, a significant decrease of the FCD, as compared to the baseline value, could be found (Fig 3).

Both surgical denervation and chemically blocking the postganglionare sympathic fibers by bupivacaine caused a change in arteriolar diameter and caused a significant decrease of the FCD, as compared to base values (Table 2, Figs 2 and 3).

## DISCUSSION

The isolated cremaster muscle model in rats allows for the direct visualization of the muscular microcirculation and, therefore, for direct investigations of the impact of topically applied agents.^11^ No alteration of the blood flow, the critical aspect of the model, could be found after preparation of the cremaster muscle in the past.^15^

After denervation of the sympathetic fibers of the cremaster muscle, the influence of the central nerve system on the vasomotion is interrupted.^16^ Directly after the surgical sectioning of the fibers, we observed a vasodilation lasting 30 minutes. These observations confirm the results of Chen et al.^17^ After 1 hour, the arteriolar diameter was unaffected, but the FCD—as parameter for tissue perfusion—decreased significantly. An increase of the FCD was observed 14 days after sectioning by Bentzer et al.^18^ Long-term follow-up studies have shown that the FCD decreases after denervation to 10% of its initial value, which could be interpreted as an adequate value for the reduced physiological requirements.^19^ In contrast to our results, in rabbits, an increase of the cutaneous arteriolar diameter after periarterial sympathectomy was found.^20^

Bupivacaine, a local anesthetic of the amino amide type, acts mainly by binding itself to the intracellular portion of sodium channels and blocking sodium influx into nerve cells, thereby blocking depolarization, as well. In our study, the A_1_- and A_2_-arterioles showed a slight vasoconstriction, the A_3_-arterioles were unaffected, and the FCD diminished significantly. This could be the result of the low level of bupivacaine concentration. Low bupivacaine concentration causes vasoconstriction, while high concentration causes vasodilation.^21^ Another reason could be the reduction of the vasodilative potency of the endothelium by the local anesthesia.^22^

The high dose of metoprolol was chosen because at that level, it blocks either the β_1_- or β_2_-receptors. The observed constriction of all arteriolar orders is caused by the blockade of the β-receptors, which have adrenergically mediated vasodilative effects. As a result of these findings, we believe that further studies are necessary. In the meantime, the use of β-receptor blockers in patients with critically perfused wounds or within microsurgical interventions should be critically evaluated, because the same effects have been found in humans.^7^

After blockade of α-receptors by phentolamine, adrenergically mediated influences should be minor, and we expected a stronger increase in arteriolar diameter than we found. This effect could have been caused by the blockade of all α-receptors, including the α_2D_-receptors, which normally cause vasodilation mediated by nitric oxide.^13^ Another study found that blockade of α-receptors caused vasodilation in humans as well.^7^ The different sensitivities of the α_1_- and α_2_-receptors play a minor role, because in the applied concentrations, the blockade could be assumed to be complete.^23^,^24^ This could explain the lack of influence on the FCD. In contrast to our results, a study on the perfusion of the ileum in rabbits showed an impairment of blood flow after the systemic administration of an α adrenoceptor blocker.^25^ This could be because the systemic administration of the blocker induces hypotension in the animal. We tried to compensate for possible systemic reactions by infusing physiological saline into the animal.

Our results show that, of the 5 groups, only the α-receptor blocker phentolamine causes vasodilation after 1 hour. A surgical or chemical interruption of the sympathetic fibers caused no increase in vasodilation. Rather, it reduced the FCD in a similar manner to topically applied metoprolol, the agent that caused the greatest decrease in diameter and the greatest impairment of FCD. These results raise the question of whether or not these frequently performed sympathetic blockades really improve microcirculation.^7^

Many patients who undergo surgical or even microsurgical intervention are on antihypertensive medications, including β-blockers, during the perioperative phase. Our results raise the question of whether β-blockers negatively influence microcirculation. Therefore, we believe that further studies are needed to investigate this potential negative side effect.

## Acknowledgment

The authors thank the Alma und Heinrich Vogelsang Foundation, Bochum, Germany, and the scientific committee, Bergmannsheil, Bochum, Germany (both foundations had no content-related involvement), for their financial support.

## Figures and Tables

**Table 1:** Weight, Rectal Temperature, Temperature of the Cremaster Muscle, and Blood Pressure of the Five Groups*

Parameter	Control	Denervation	Bupivacaine	Metoprolol	Phentolamine
Animal weight, g	182.7 ± 3.0	185.3 ± 1.6	186.9 ± 1.9	187.4 ± 2.4	186.8 ± 1.8
Rectal temperature, 0 min, °C	35.6 ± 0.2	35.7 ± 0.2	35.7 ± 0.2	36.0 ± 0.2	35.9 ± 0.2
Rectal temperature, 60 min, °C	35.6 ± 0.2	35.4 ± 0.3	35.4 ± 0.4	35.8 ± 0.2	35.8 ± 0.2
Muscle temperature, 0 min, °C	32.2 ± 0.2	32.9 ± 0.2	33.1 ± 0.2	32.9 ± 0.2	32.8 ± 0.3
Muscle temperature, 60 min, °C	32.3 ± 0.3	32.4 ± 0.3	32.6 ± 0.3	32.6 ± 0.2	32.7 ± 0.2
Blood pressure,† 0 min	106.8 ± 1.6	95.2 ± 6.6	95.5 ± 8.3	106.3 ± 1.8	102.8 ± 2.7
Blood pressure,† 60 min	99.8 ± 2.2	95.1 ± 1.7	96.3 ± 1.8	95.5 ± 1.8	98.3 ± 2.2

*Data is given in mean and standard error of mean; 0 = baseline value.

†Blood pressure measured within the carotid arteries.

**Table 2:** Diameter and Functional Capillary Density of the 5 Groups*

Parameter	Time	Control	Denervation	Bupivacaine	Metoprolol	Phentolamine
A_1_-arterioles [µm]	0	80.73 ± 4.25	85.16 ± 3.21	90.10 ± 4.03	79.17 ± 3.38	76.56 ± 6.89
	60	86.98 ± 5.98	77.87 ± 4.68	80.73 ± 7.32	72.92 ± 6.27	100.00 ± 12.02
A_2_-arterioles [µm]	0	60.94 ± 3.28	67.66 ± 5.08	66.15 ± 3.56	64.58 ± 7.74	51.04 ± 4.13
	60	59.90 ± 4.52	64.58 ± 5.20	59.90 ± 5.13	54.17 ± 7.93	64.58 ± 8.01
A_3_-arterioles [µm]	0	39.58 ± 3.01	38.80 ± 4.41	36.98 ± 3.39	35.94 ± 4.29	29.69 ± 2.89
	60	40.10 ± 3.80	40.00 ± 4.04	37.50 ± 4.00	28.65 ± 3.96†	36.98 ± 3.48
FCD [*n*/0.22 mm^2^]	0	6.38 ± 0.51	9.11 ± 0.52	11.60 ± 0.63	9.99 ± 0.73	11.21 ± 0.98
	60	6.41 ± 0.80	6.64 ± 0.93†	4.55 ± 1.10†	4.09 ± 0.95†	10.87 ± 1.29

*Data is given in mean and SEM; FCD indicates functional capillary density.

†*P* < .05 versus baseline value;
